# Prevalence and multivariable factors associated with inadvertent intraoperative hypothermia in video-assisted thoracoscopic surgery: a single-center retrospective study

**DOI:** 10.1186/s12871-020-0953-x

**Published:** 2020-01-28

**Authors:** Yinan Li, Hansheng Liang, Yi Feng

**Affiliations:** Department of Anesthesia, Peking University People’s Hospital, Beijing, 100044 China

**Keywords:** Hypothermia, Video-assisted thoracoscopic surgery (VATS), Incidence, Risk factors, Paravertebral block (PVB)

## Abstract

**Background:**

Inadvertent intraoperative hypothermia increases the risk of adverse events, but its related risk factors have not been defined in video-assisted thoracoscopic surgery (VATS). This study aimed at analyzing the prevalence and factors related to inadvertent intraoperative hypothermia in adults undergoing elective VATS under general anesthesia.

**Methods:**

This was a retrospective study using data from the Peking University People’s Hospital from January through December, 2018. Data were collected on age, sex, height, weight, American Society of Anesthesiologists physical status, the duration of preparation and surgery, timing of surgery, surgery types, anesthesia types, intraoperative core temperature and the length of stay (LOS) in the hospital from the electronic database in our center. Patients were covered with a cotton blanket preoperatively and the surgical draping during the operation. A circulation-water warming mattress set to 38 °C were placed under the body of the patients. Inadvertent intraoperative hypothermia was identified as a core temperature monitored in nasopharynx < 36 °C. Multivariate logistic regression analysis was used to identify independent risk factors of hypothermia.

**Results:**

We found that 72.7% (95% CI 70.5 to 75.0%) of 1467 adult patients who underwent VATS suffered hypothermia during surgery. The factors associated with inadvertent intraoperative hypothermia included age (OR = 1.23, 95% CI 1.11 to 1.36, *p* < 0.001), BMI (OR = 1.83, 95% CI 1.43 to 2.35, *p* < 0.001), the duration of preparation (OR = 1.01, 95% CI 1.00 to 1.02, *p* = 0.014), the duration of surgery (OR = 2.10, 95% CI 1.63 to 2.70, *p* < 0.001), timing of surgery (OR = 1.64, 95% CI 1.28 to 2.12, *p* < 0.001), ambient temperature in the operating room (OR = 0.67, 95% CI 0.53 to 0.85, *p* = 0.001) and general anesthesia combined with paravertebral block after induction of anesthesia (OR = 2.30, 95% CI 1.31 to 4.03, *p* = 0.004). The average LOS in the hospital in the hypothermia group and the normothemic group was 9 days and 8 days, respectively (*p* < 0.001).

**Conclusions:**

We highlight the high prevalence of inadvertent intraoperative hypothermia during elective VATS and identify key risk factors including age, duration of surgery more than 2 h, surgery in the morning and general anesthesia combined with paravertebral block (PVB) after intubation. We also find that hypothermia did prolong the LOS in the hospital.

## Background

Hypothermia can be divided into therapeutic hypothermia and inadvertent hypothermia. Inadvertent intraoperative hypothermia, a recognized common perioperative complication, is defined as a core temperature (Tc) < 36 °C with an incidence as high as 70% in adults undergoing elective and emergency surgery [1]. Meanwhile, hypothermia might be associated with increased risk of several different kinds of adverse events. For instance, morbid cardiac events and ventricular tachycardia, blood loss, wound infections, delayed post-anesthetic recovery time and hospitalization, and postoperative cognitive dysfunction [2–6].

Compared to open thoracic surgery, video-assisted thoracoscopic surgery (VATS) is minimally invasive and lowers the exposure of the thoracic viscera to the environment; however, VATS is still vulnerable to perioperative hypothermia [7]. Accordingly, we performed a retrospective study to determine the incidence and factors associated with inadvertent intraoperative hypothermia in adult patients during elective VATS under general anesthesia combined with paravertebral block (PVB) or not.

## Methods

This single-center retrospective study was approved by the Ethical Review Committee of Peking University People’s Hospital (Beijing, China) and informed consent was waived (No.2019PHB135–01). We retrieved electronic medical information from databases in our center and reviewed data from adult patients who underwent elective VATS under general anesthesia or combined with PVB in the Peking University People’s Hospital from January through December, 2018. Patients < 18 years were excluded. Patients who underwent thoracotomy, laparotomy, hyperthermia intrapleural chemotherapy, emergency surgery, non-surgical or cardiac procedures, or under local anesthesia were excluded. Those lacking ambient temperature records or temperature monitoring were also excluded.

We recorded information in an electronic anesthetic documentation system certified by the Healthcare Information and Management Systems Society and rated to Electronic Medical Records Adoption Model stage 7 (HIMSS EMRAM7) in our center in 2014. Our data were all obtained from the electronic database on the server. We collected demographic data including age, sex, height and weight (used to calculate the body mass index, BMI and body surface area, BSA) and American Society of Anesthesiologists physical status (ASA). Clinical data including duration of preparation and surgery, time of surgery (morning or afternoon), types of surgery (wedge resection, lobectomy, mediastinoscopy and others), modes of anesthesia (general anesthesia or general anesthesia combined with PVB) and intraoperative core temperature were collected from the anesthetic documentation. The total time in the operating room (OR) including the preparation time from patients arriving in the OR to the start of surgery, the duration of surgery from surgical incision to the last suture, and the interval from the end of surgery to patients leaving the OR were recorded. The length of stay (LOS) in the hospital was also collected. The ambient temperature and relative humidity in the OR were obtained from the records of the hospital’s control center. Patients received routine nasopharyngeal temperature monitoring as intraoperative core temperature in our study. All patients were covered with an unheated cotton blanket preoperatively and the surgical draping during operation for thermal insulation. A circulation-water warming mattress under the body of the patients was set to 38 °C intraoperatively. We didn’t use fluid warming or forced air warming device until the core temperature dropped to 35.5 °C. Inadvertent intraoperative hypothermia was defined as a core temperature (Tc) below 36 °C during the intraoperative phase while normothemic referred to a temperature of Tc ≥36 °C throughout the operation. Although Our monitoring system automatically recorded the temperature data every 10s, we collected nasopharyngeal temperature data at intervals of 5 min, and determine the occurrence of hypothermia with all records less than 36 °C within 5 min after the start of the 5 min-data less than 36 °C in order to exclude “false” hypothermia due to factors such as equipment problems or accidental probe displacement.

Continuous variables are shown as the mean (standard deviation) in the normal distribution or the median [interquartile range] where abnormally distributed data were encountered. Frequency (percentages) distributions are shown in categorical variables. Univariate logistic regression analyses were used to explore the influencing factors for inadvertent intraoperative hypothermia, and brought variables with *p-*value less than 0.1 into multivariate logistic regression analyses with backward stepwise regression. The results were expressed as odds ratios with a 95% confidence interval. All statistical analyses were carried out by IBM SPSS, version 22.0. A *p-*value less than 0.05 was considered statistically significant.

## Results

**Fig. 1:**
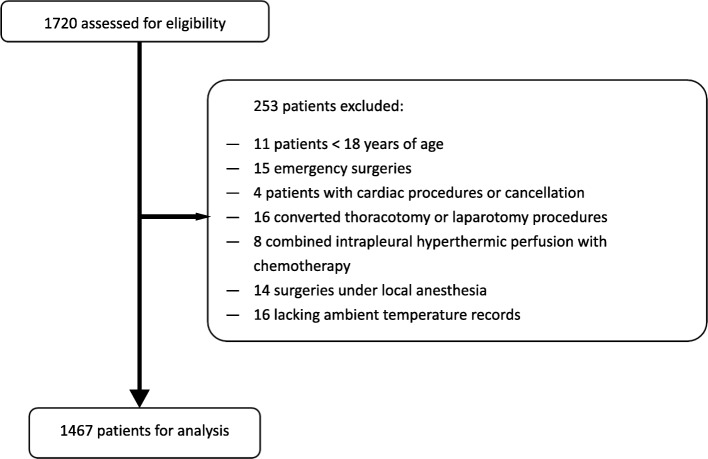
Study Flow

After retrieving data from 1720 patients, 1467 were finally analyzed. The flow diagram is shown in Fig. 1 (page 17). Patients ranged 51–66 years of age (58 yrs. [51-66 yrs]), and 50.4% were overweight (BMI ≥ 24 kg/m^2^, *n* = 740). Most patients underwent lobectomy (*n* = 856, 58.4%) and were ASA II class (*n* = 1113, 75.9%). The mean ambient temperature and humidity was 22.6 °C (standard deviation 0.5 °C) and 41% (standard deviation 9%), respectively. PVB was widely applied in our study, with 73.6% patients (*n* = 1079) were applied either before or after induction of anesthesia. More than half of the patients (*n* = 778, 53%) had surgery in the morning (from 8 am to 2 pm) with the remainder having surgery in the afternoon (from 2 pm to 8 pm). Statistical differences were observed for all variables excluding sex (*p* = 0.057) and ASA (*p* = 0.516) between the two groups. And the average LOS in the hospital in the hypothermia group and the normothemic group was 9 days and 8 days, respectively (*p* < 0.001) (Table 1, page 13 to 14). Of the 1467 cases, only one in the hypothermia group died before discharge due to postoperative myocardial infarction.

**Table 1:** Demographic and clinical characteristics of the patients (*n* = 1467)

Variable	Hypothermia (*n* = 1067)	Normothermia (*n* = 400)	*P* value
Age (yr)	60 [53–67]	56 [48–64]	< 0.001*
Sex			0.057
female	577 (54.1%)	194 (48.5%)	
male	490 (45.9%)	206 (51.5%)	
BMI (kg/m^2^)	23.81 [21.80–25.80]	24.75 [22.68–26.89]	< 0.001*
< 24	563 (52.8%)	164 (41.0%)	
≥ 24	504 (47.2%)	236 (59.0%)	
BSA (m^2^)	1.80 [1.69–1.92]	1.86 [1.73–2.01]	< 0.001*
≤ 1.83	626 (58.7%)	186 (46.5%)	
> 1.83	441 (41.3%)	214 (53.5%)	
ASA			0.516
I	175 (16.4%)	73 (18.3%)	
II	811 (76.0%)	302 (75.5%)	
III	81 (7.6%)	25 (6.3%)	
Duration of Preparation (min)	60 [49–72]	54 [46–66]	< 0.001*
≤ 1 h	551 (51.6%)	263 (65.8%)	
> 1 h	516 (48.4%)	137 (34.3%)	
Time of Surgery			< 0.001*
Morning	616 (57.7%)	162 (40.5%)	
Afternoon	451 (42.3%)	238 (59.5%)	
Duration of Surgery (min)	122 [94–156]	100.5 [74–130]	< 0.001*
≤ 2 h	511 (47.9%)	267 (66.8%)	
> 2 h	556 (52.1%)	133 (33.3%)	
Time in OR (min)	201 [168–237]	174 [140.5–210]	< 0.001*
≤ 3 h	378 (35.4%)	227 (56.8%)	
> 3 h	689 (64.6%)	173 (43.3%)	
OR temperature (°C)	22.6 [22.3–23.0]	22.7 [22.4–23.1]	< 0.001*
OR humidity (%)	40 [36–51]	39 [36–48]	< 0.001*
Type of Surgery			< 0.001*
wedge resection	281 (26.3%)	153 (38.3%)	
lobectomy	667 (62.5%)	189 (47.3%)	
mediastinum	98 (9.2%)	46 (11.5%)	
others	21 (2.0%)	12 (3.0%)	
Type of Anesthesia			< 0.001*
general anesthesia alone	289 (27.1%)	99 (24.8%)	
PVB before intubation	632 (59.2%)	283 (70.8%)	
PVB after intubation	146 (13.7%)	18 (4.5%)	
LOS in the hospital (day)	9 [7–11]	8 [7–10]	< 0.001*

Values are shown as the median [interquartile range] or number (%), analyzed using a Mann-Whitney U test or chi-square test

*OR* operating room, *BMI* body mass index, *BSA* body surface area, BSA = 0.0061*height (cm) + 0.0124*weight (kg)-0.0099; *ASA* American Society of Anesthesiologists physical status, *PVB* paravertebral block, *LOS* length of stay

Duration of preparation refers to the interval from arrival in the OR to incision

Time in the OR refers to minutes from arrival in the OR to leaving the OR

Among the total population, 1067 (72.7, 95% CI 70.5 to 75.0%) patients suffered from intraoperative hypothermia. According to the univariate logistic regression (Table 2, page 15), *p-*values ≤0.1 were analyzed by multivariate logistic regression, and ultimately, there were seven variables independently related to inadvertent intraoperative hypothermia (Table 3, page 16). High ambient temperature (OR = 0.67, 95% CI 0.53 to 0.85, *p* = 0.001) in the OR was protective against hypothermia; and age (OR = 1.23, 95% CI 1.11 to 1.36, *p* < 0.001), BMI < 24 kg/m^2^ (OR = 1.83, 95% CI 1.43 to 2.35, *p* < 0.001), duration of preparation (OR = 1.01, 95% CI 1.00 to 1.02, *p* = 0.014), duration of surgery more than 2 h (OR = 2.10, 95% CI 1.63 to 2.70, *p* < 0.001), undergoing surgery in the morning (OR = 1.64, 95% CI 1.28 to 2.12, *p* < 0.001) and general anesthesia combined with PVB after intubation (OR = 2.30, 95% CI 1.31 to 4.03, *p* = 0.004) were independent risk factors for hypothermia.

**Table 2:** Univariate logistic regression for inadvertent intraoperative hypothermia rates

	*β*	Wald	Odds ratio	95% confidence interval		*P* value
Age (per decade)	.256	27.188	1.292	1.173	1.423	.000*
Female	.223	3.623	1.250	.993	1.574	.057
BMI (kg/m^2^)	−.100	26.662	.905	.871	.940	.000*
< 24	.475	15.985	1.607	1.274	2.029	.000*
BSA (m^2^)	−2.101	36.946	.122	.062	.241	.000*
≤ 1.83	.491	17.293	1.633	1.296	2.058	.000*
ASA						
I	/	1.319	/	/	/	.517
II	.114	.538	1.120	.827	1.517	.463
III	.301	1.265	1.352	.799	2.285	.261
Duration of Preparation (min)	.019	23.995	1.019	1.012	1.027	.000*
> 1 h	.587	23.160	1.798	1.416	2.283	.000*
Surgery in the morning	.696	34.122	2.007	1.588	2.535	.000*
Duration of Surgery (min)	.009	45.492	1.009	1.007	1.012	.000*
> 2 h	.781	40.642	2.184	1.718	2.777	.000*
Time in OR (min)	.008	46.178	1.008	1.006	1.010	.000*
> 3 h	.872	53.239	2.392	1.892	3.023	.000*
OR temperature (°C)	−.388	11.685	.678	.543	.847	.001*
OR humidity (%)	.026	11.132	1.026	1.011	1.042	.001*
Type of Surgery						
lobectomy	/	28.149	/	/	/	.000
wedge resection	−.653	25.264	.520	.403	.671	.000*
mediastinum	−.505	6.577	.604	.410	.888	.010*
others	−.701	3.572	.496	.240	1.026	.590
Type of Anesthesia						
general anesthesia alone	/	26.221	/	/	/	.000
PVB before intubation	−.268	3.842	.765	.585	1.000	.050*
PVB after intubation	1.022	13.747	2.779	1.619	4.769	.000*

*BMI* body mass index, *BSA* body surface area, BSA = 0.0061*height (cm) + 0.0124*weight (kg)-0.0099; *ASA* American Society of Anesthesiologists physical status, *OR* operating room, *PVB* paravertebral block

Time in OR refers to minutes from arrival in OR to leaving OR

**Table 3:** Multivariate logistic regression for inadvertent intraoperative hypothermia rates

	*β*	Wald	Adjusted odds ratio	95% confidence interval		*P* value
Age (per decade)	.206	15.898	1.229	1.111	1.360	.000*
BMI < 24 kg/m^2^	.606	23.351	1.833	1.434	2.345	.000*
Surgery in the morning	.497	14.915	1.644	1.278	2.117	.000*
Duration of Preparation (min)	.010	5.995	1.011	1.002	1.019	.014*
Duration of Surgery> 2 h	.742	33.426	2.099	1.633	2.699	.000*
OR temperature (°C)	−.401	11.243	.670	.530	.847	.001*
Type of Anesthesia						
general anesthesia alone	/	10.797	/	/	/	.005
PVB before intubation	−.084	.309	.920	.685	1.235	.579
PVB after intubation	.832	8.441	2.298	1.311	4.029	.004*

Method = Backward Stepwise (Likelihood Ratio)

*BMI* body mass index, *OR* operating room, *PVB* paravertebral block

*p-value less than 0.05 was considered statistically significant

## Discussion

In the current study, the prevalence of inadvertent intraoperative hypothermia in VATS was 72.7%, with related risk factors including older age, longer preparation and surgery time, lower ambient temperature, receiving surgery in the morning and general anesthesia combined with PVB after intubation. Being overweight was a protective factor.

In a previous report, the overall incidence of hypothermia was 64.3% during thoracic surgery. We included a more strict definition of hypothermia ≤36 °C and our study cohort had a lower mean BMI than previous studies, explaining such discrepancies [8]. Being overweight is protective owing to the body fat maintaining heat balance through triggering vasoconstriction early when core temperatures decreased in obese patients [9]. Other studies divided patients into hypothermic groups at the end of surgery, which of lower incidence due to the active warming measures performed intraoperatively [10].

It was not surprised that intraoperative hypothermia is more likely to occur at an advanced age, at low room temperature, or during prolonged surgery. This was consistent with patients receiving abdominal surgery under general anesthesia [11]. Previous studies demonstrated that thermoregulatory vasoconstriction threshold is reduced in elderly patients under general anesthesia [12]. The duration of surgery was related to the decline of Tc, in agreement with the results of Suneerat et al. [13]. A longer duration of surgery results in a longer intrathoracic organ exposure time and a longer duration of anesthesia. Both general and neuraxial anesthesia could impair thermoregulatory control [14]. In this study, ambient temperatures were positively associated with core temperatures. This was in accordance with a previous study which demonstrated a higher temperature in the operating room being a strong protective factor against hypothermia [13]. However, if patients used active warming strategies such as forced-air blankets, the effects of intraoperative ambient temperatures on hypothermia were mild [15].

A significant finding of this retrospective study was that the data analysis showed timing of surgery or paravertebral block were also important factors influencing intraoperative hypothermia. Compared to surgery performed in the afternoon, the risk of intraoperative hypothermia was 1.6 fold higher than in the morning. This may be due to the epigenetic clock function in body temperature as reported in different seasons in a previous study. According to a study investigating individual body temperature, diurnal variation in temperature peak at 4 pm compared to 12 pm [16]. Furthermore, those undergoing surgery in the afternoon provide perioperative myocardial protection [17]. Hence, it is beneficial to perform surgery in the afternoon compared to in the morning to regarding in reduction of reduce the occurrence of hypothermia.

This is the first documented report to establish relationships between PVB and hypothermia. There were no statistical differences between general anesthesia with PVB and general anesthesia alone related to hypothermia in univariate logistic regression. According to the blocking time, ultrasound-guided PVB was further divided into the pre-induction PVB group and the post-induction PVB group. Patients who received ultrasound-guided PVB first and then underwent general anesthesia were referred to as pre-induction PVB, while patients who received ultrasound-guided PVB after general anesthesia were referred to as post-induction PVB. After regrouping PVB according to blocking time, compared with general anesthesia alone, the risk of intraoperative hypothermia was 2.3 fold higher in patients undergoing general anesthesia combined with post-induction PVB by multivariate logistic regression analysis. Compared to the epidural anesthesia (EA), PVB blocks ipsilateral sympathetic nerves and is associated with a reduction in hypotension [18]. As known, EA leads to hypothermia through its ability to redistribute heat [19]. PVB may therefore promote hypothermia through the same mechanism. However, it may have a “hypothermic preconditioning” function which differs from EA due to its unilateral sympathetic block. PVB may neutralize variations in Tc by enhancing thermogenesis before general anesthesia impairing thermoregulatory control. Further randomized controlled studies are required to establish the relationship between PVB and hypothermia.

There were some limitations in this study. First, only the relationship between hypothermia and variations were analyzed but the etiology associated was not discussed. Then, the core temperature data of PACU cannot be obtained retrospectively, so we did not analyze the correlation between intraoperative core temperature and PACU temperature. And a further limitation was the study being single-centered, limiting its external validity.

## Conclusions

The prevalence of inadvertent intraoperative hypothermia in adult patients during elective VATS under general anesthesia is high. And we find that hypothermia did prolong the length of stay. Being overweight prevented patients from hypothermia, while independent risk factors associated with hypothermia were old age, a duration of surgery more than 2 h, morning surgery and general anesthesia combined with PVB after intubation. Advanced prospective studies are now required evaluate the relationship between PVB and inadvertent intraoperative hypothermia.

## Data Availability

The datasets used and analysed during the current study are available from the corresponding author on reasonable request.

## Abbreviations

ASA: American Society of Anesthesiologists physical status
BMI: Body mass index
EA: Epidural anesthesia
HIMSS EMRAM7: the Healthcare Information and Management Systems Society and rated to Electronic Medical Records Adoption Model stage 7
OR: Operating room
PVB: Paravertebral block
Tc: Core temperature
VATS: Video-assisted thoracoscopic surgery
